# Platelet CLEC2-Podoplanin Axis as a Promising Target for Oral Cancer Treatment

**DOI:** 10.3389/fimmu.2021.807600

**Published:** 2021-12-15

**Authors:** Byeong-Oh Hwang, Se-Young Park, Eunae Sandra Cho, Xianglan Zhang, Sun Kyoung Lee, Hyung-Joon Ahn, Kyung-Soo Chun, Won-Yoon Chung, Na-Young Song

**Affiliations:** ^1^ Department of Applied Life Science, The Graduate School, Yonsei University, Seoul, South Korea; ^2^ BK21 Four Project, Yonsei University College of Dentistry, Seoul, South Korea; ^3^ Department of Oral Biology, Yonsei University College of Dentistry, Seoul, South Korea; ^4^ Department of Oral Pathology, Yonsei University College of Dentistry, Seoul, South Korea; ^5^ Oral Cancer Research Institute, Yonsei University College of Dentistry, Seoul, South Korea; ^6^ Department of Pathology, Yanbian University Hospital, Yanji City, China; ^7^ Department of Orofacial Pain and Oral Medicine, Dental Hospital, Yonsei University College of Dentistry, Seoul, South Korea; ^8^ College of Pharmacy, Keimyung University, Daegu, South Korea

**Keywords:** platelets, tumor cell-induced platelet aggregation (TCIPA), CLEC2, PDPN, ezrin/radixin/moesin (ERM), oral cancer

## Abstract

Cancer tissues are not just simple masses of malignant cells, but rather complex and heterogeneous collections of cellular and even non-cellular components, such as endothelial cells, stromal cells, immune cells, and collagens, referred to as tumor microenvironment (TME). These multiple players in the TME develop dynamic interactions with each other, which determines the characteristics of the tumor. Platelets are the smallest cells in the bloodstream and primarily regulate blood coagulation and hemostasis. Notably, cancer patients often show thrombocytosis, a status of an increased platelet number in the bloodstream, as well as the platelet infiltration into the tumor stroma, which contributes to cancer promotion and progression. Thus, platelets function as one of the important stromal components in the TME, emerging as a promising chemotherapeutic target. However, the use of traditional antiplatelet agents, such as aspirin, has limitations mainly due to increased bleeding complications. This requires to implement new strategies to target platelets for anti-cancer effects. In oral squamous cell carcinoma (OSCC) patients, both high platelet counts and low tumor-stromal ratio (high stroma) are strongly correlated with increased metastasis and poor prognosis. OSCC tends to invade adjacent tissues and bones and spread to the lymph nodes for distant metastasis, which is a huge hurdle for OSCC treatment in spite of relatively easy access for visual examination of precancerous lesions in the oral cavity. Therefore, locoregional control of the primary tumor is crucial for OSCC treatment. Similar to thrombocytosis, higher expression of podoplanin (PDPN) has been suggested as a predictive marker for higher frequency of lymph node metastasis of OSCC. Cumulative evidence supports that platelets can directly interact with PDPN-expressing cancer cells *via* C-type lectin-like receptor 2 (CLEC2), contributing to cancer cell invasion and metastasis. Thus, the platelet CLEC2-PDPN axis could be a pinpoint target to inhibit interaction between platelets and OSCC, avoiding undesirable side effects. Here, we will review the role of platelets in cancer, particularly focusing on CLEC2-PDPN interaction, and will assess their potentials as therapeutic targets for OSCC treatment.

## Introduction

Oral squamous cell carcinoma (OSCC) is the most prevalent type of head and neck malignancies that occur in oral cavity, salivary gland, pharynx, larynx, nasal cavity, thyroid, and bone (1). Unlike the other types of cancers, OSCC usually arises from the body part that is easily accessible for visual examinations. Despite this advantage in detection of precancerous lesions, most of the OSCC patients are not diagnosed until the advanced stages with metastasis, which is attributed to low overall survival rates (2). Oral mucosa contains a connective tissue enriched with type I collagen that is synthesized by stromal cells (3). Desmoplasia, a status of the excessive growth of the stromal tissue, is closely associated with OSCC (4, 5). In OSCC patients, stroma-rich tumors are more aggressive and metastatic than stroma-poor tumors, finally contributing to the poor survival rates (6, 7). The activated tumor stroma can supply a variety of growth factors and cytokines that induces cancer cell proliferation as well as extracellular matrix (ECM) remodeling (5, 8). In support of the tumor stroma, OSCC cells tend to invade adjacent tissues, such as bones, and spread to the lymph nodes (9). This locoregional characteristic of OSCC is the primary cause of treatment failure (10). Thus, how to control the local and distal metastasis is crucial for successful treatment and better prognosis in OSCC patients.

Platelets, the smallest cells in blood circulation, play a major role in blood coagulation and hemostasis (11–13). In addition to their primary physiological functions, platelets are profoundly involved in cancer promotion and progression (14, 15). Recently, it has been reported that platelets can infiltrate into the tumor stroma in colorectal and pancreatic cancer patients (16–18). As a part of the tumor stromal components, platelets crosstalk with cancer cells either directly or indirectly, promoting invasion and metastasis (19–21). For the physical interaction, C-type lectin-like receptor 2 (CLEC2) and podoplanin (PDPN) are suggested as the key molecular links expressed in platelets and tumors, respectively (22). Moreover, cancer cells activate and educate platelets, thus the bilateral interaction between platelets and cancer can further promote tumorigenesis, creating a positive feedback loop (23). Notably, OSCC patients often show increased platelet counts, which is strongly associated with poor prognosis (24, 25). Thus, platelets are emerging as an important target for chemotherapy in OSCC patients.

Aspirin, a representative antiplatelet agent, is well known to protect against carcinogenesis (26–28). Aspirin irreversibly inhibits both cyclooxygenase-1 (COX-1) and COX-2, reducing synthesis of prostaglandins and thromboxanes responsible for inflammation and platelet aggregation (27). Despite its chemopreventive effect, a daily use of low-dose aspirin frequently causes adverse complications, primarily increased bleeding risk (29, 30). Thus, instead of using traditional antiplatelet agents, the pinpoint targeting of the platelet-tumor cell interaction would be a more precise and effective strategy for OSCC treatment, avoiding undesirable harmful effects. In this regards, we will highlight the role of platelets in carcinogenesis and OSCC, particularly focusing on the physical interaction between platelets and tumors *via* the CLEC2-PDPN axis.

## Roles of Platelets in Cancer

### Thrombocytosis in Cancer Patients

Platelets are anucleated cells originated from megakaryocytes in the bone marrow and abundant in healthy individual 150,000~400,000 per microliter of blood (11–13). In spite of lack of genomic DNA, platelets release plenty of granular ingredients, such as platelet-derived growth factor (PDGF), transforming growth factor β (TGFβ), stromal cell-derived factor-1 (SDF-1), and serotonin, which contributes to signal transduction in nearby cells (31). Cancer is often associated with thrombocytosis, a status of an abnormal elevation of platelet counts, which shows a positive correlation with worse outcomes in many types of cancers (24, 32–34). High platelet counts are involved with development of venous thromboembolism (VTE) in cancer patients, the second leading cause of cancer death (35–38). Besides an increased risk of VTE, thrombocytosis is associated with cancer mortality by accelerating tumor promotion and progression as well (39–41). In mice bearing tumors, platelet transfusion induced the blood platelet counts as well as tumor growth, while reducing the survival rates (37, 42). Thus, the platelet counts have long been considered as a valuable prognostic marker in cancer patients.

It has been reported that inflammatory cytokines, such as interleukin-6 (IL-6), are highly associated with thrombocytosis in cancer patients (33, 43). IL-6 can stimulate platelet production through inducing thrombopoietin (33, 44). In murine colitis model, colitis-induced wild type (WT) mice showed thrombocytosis and platelet aggregation, which were absent in IL-6-deficient mice (45). Moreover, neutralization of IL-6 led to reduction of platelet counts and tumor growth in the mouse ovarian cancer model (33). Thus, IL-6 inhibitors might be utilized to mitigate cancer-associated thrombocytosis (46). However, anti-IL-6 treatments need meticulous assessment, regarding that IL-6 pleiotropically functions in immune system (47, 48).

### Platelets as a Part of Stromal Components in Tumor Microenvironment

Tumor tissues are not just simple masses of malignant cells, but rather complex and heterogeneous collections of cellular and even non-cellular components, referred to as tumor microenvironment (TME) (49). The multiple players in the TME develop dynamic interactions with each other, which determines the characteristics of the tumor (50). The non-cellular parts of the TME comprise primarily the ECM, a three-dimensional scaffold that contains collagens, proteoglycans, and fibronectins (51). The acellular ECM is crucial for providing mechanical (structural) and biochemical (nutritional) supports to cellular components in the TME (52). The cellular players in the TME can be largely divided into stromal cells and tumor-infiltrating immune cells. The tumor stroma is a heterogeneous population of distinct types of cells, including fibroblasts and endothelial cells (53). Among them, cancer-associated fibroblasts (CAFs) are the most abundant type of the stromal cells in TME that display enhanced expression of the signature proteins, including α-smooth muscle actin and PDGF receptors (54). Moreover, the TME contains a broad spectrum of immune cells, such as tumor-associated macrophages, tumor-associated neutrophils, and regulatory T cells. Notably, the infiltration of platelets into the tumor stroma has been observed in cancer patients (18, 55, 56), along with increased blood platelet counts (24, 32–34). Tumor-infiltrating platelets can interact with other stromal players of TME, contributing to tumor promotion and progression (57). Miyashita et al. have found that CAFs were surrounded by platelets in almost half of the pancreatic cancer patients (58). Platelet-derived factors, like TGFβ, PDGF, and SDF-1, can stimulate recruitment and activation of CAFs in the TME (59–62). Platelets also accommodate various angiogenesis regulators, which can turn on local angiogenesis in the TME (63). Depletion of tumor-infiltrating platelets showed impaired tumor blood vessel structures in mice (64). Moreover, it has been reported that fusion between platelets and endothelial cells promotes cancer metastasis by facilitating adhesion of tumor and endothelial cells (65). In consistent, the intratumoral accumulation of platelets are related to tumor progression (18, 55, 56). These investigations support that platelets function as a crucial stromal component in the TME through vigorous interplay with other members.

### Platelets in Cancer Invasion and Metastasis

Metastasis is a multi-step process, including local invasion, intravasation, and colonization at the distal sites (66). Invading cancer cells undergo dramatic alterations in their morphology and phenotypes, such as epithelial-to-mesenchymal transition (EMT), which is accompanied by remodeling of the ECM (66). As a poor prognostic indicator, thrombocytosis is associated with lymph node metastasis and invasion in cancer patients (24, 67). In consistent, platelet transfusion significantly enhanced metastasis of cancer cells in the murine experimental models (15, 68). However, platelet decoys bound to tumor cells as effectively as normal intact platelets and inhibited thrombosis and metastatic tumor formation, further supporting the role of platelets in metastasis (69). Of note, platelets are frequently detected at the invasive front where both EMT and ECM remodeling occur actively (70). Platelets contain about 40% of TGFβ found in the peripheral blood plasma, which plays a crucial role in cancer cell invasion (71). Co-culture with platelets remarkably enhanced invasiveness and EMT process of cancer cells in a TGFβ-dependent manner (72, 73). Platelet-specific *Tgfb1*-deficient mice showed reduction in tumor growth and platelet extravasation, compared to WT mice (74). Moreover, various types of matrix metalloproteinases (MMPs) responsible for ECM degradation are stored in the resting platelets and released upon stimulation, such as cancer cell-induced aggregation (35, 75, 76). Platelets upregulate production of MMPs in cancer cells as well as fibroblasts, accelerating invasion of cancer cells (77–79). These data suggest that platelets can change TME through their releasates, such as TGFβ and MMPs, conferring cancer cells invasive capability and metastatic potential. In addition, direct contact with platelets can promote invasion and metastasis of cancer cells *in vitro* and *in vivo* (19, 80).

Platelets can promote metastasis through interaction with other cells in the bloodstream as well, like in the TME. Platelets rapidly adhere to circulating cancer cells in the blood, protecting tumors from immune surveillance (41, 81). Natural killer (NK) and CD8 T cells are cytotoxic lymphocytes that play a central role in cancer immunosurveillance (82). Once tumors are coated by platelets, platelets inhibit NK cell-mediated antitumor activity through downregulating tumor cell NK2D expression by TGFβ and inducing pseudoexpression of immunomodulating molecules, such as MHC I and GITR (83–85). Moreover, platelet-derived factors, such as TGFβ and programmed death-ligand 1 (PD-L1), suppressed the cytotoxic antitumor T cell immunity in the mouse cancer models (86–88). Taken together, these data suggest that platelets facilitate tumor immune escape by surrounding cancer cells in the bloodstream, thus, the platelet-camouflaged cancer cells safely migrate to the metastatic sites. Of note, co-incubation with platelets protected cancer cells against anoikis, implying that platelets enhance anchorage-independent survival of circulating tumor cells in the bloodstream (15).

### Platelets as a Potential Target for OSCC Treatment

Similar to other types of cancer patients, increased platelet counts are significantly correlated with poor prognosis in OSCC patients (25, 89). Based on the analysis of relationship between platelet counts and disease progression in a total of 253 OSCC patients, thrombocytosis was associated with lymph node metastasis as well as distant metastasis (90). Along with metastasis, advanced OSCC often shows invasion into the facial bones, due to close anatomical relationship (91, 92). The bone invasion causes severe pains, greatly lowering the quality of life and the survival rates in OSCC patients (91, 93). Notably, platelet aggregation plays a critical role in tumor-associated bone destruction (94). In line with that, the pharmacological inhibition of platelet aggregation reduced bone metastasis in the murine cancer model (95). Platelet-secreted lysophosphatidic acid is thought to be one of the primary mediators in platelet-promoted bone invasion and metastasis (96, 97). Taken together, platelets can facilitate bone invasion through direct contact with tumor as well as their releasates. In OSCC, bone destruction and invasion are closely related to TGFβ signaling pathway (98, 99). Considering that platelets store most of the plasma TGFβ, it is plausible that platelets aggravate invasion of OSCC, and thus further pre-clinical and clinical investigations will shed light on a noble would be novel strategies for OSCC treatment.

## Interaction Between Platelets and Cancer: CLEC2-PDPN-ERM Axis

### PDPN in Cancer and Platelet Aggregation

PDPN is a type I transmembrane glycoprotein expressed in kidney podocytes, skeletal muscles, lungs, hearts, myofibroblasts, osteoblasts, mesothelial cells, and lymphatic endothelial cells (100). PDPN knockout mice die shortly after birth due to an impaired respiratory system (101). These mice also show defects in the lymphatic vasculature, disorganization of spleen, and lack of lymph nodes (102, 103). PDPN is thus an important regulator in the normal organogenesis and development processes.

Upregulation of PDPN has been observed in a variety of human cancers, including brain cancer, breast cancer, lung cancer, and mesothelioma, which is associated with poor prognosis (104–107). In athymic nude mice, injection of PDPN-overexpressing cancer cells generated bigger tumors, while silencing of PDPN suppressed tumor growth (108). Moreover, PDPN-high tumors exhibited increased peritumoral lymphangiogenesis, invasiveness, migratory ability, and metastasis, implying a pro-tumorigenic role of PDPN (109–112). Notably, it has been reported that PDPN expression is elevated at the leading edge of tumor tissues, which promotes cell surface extension and cell motility in keratinocytes (111, 113). In the two-stage skin carcinogenesis model, epidermal ablation of PDPN reduced tumor growth and invasion (109). Overall, these data suggest that PDPN confers cancer cells survival benefits, promoting tumor growth, invasion, and metastasis.

Interestingly, PDPN-overexpressing cancer cells evoke platelet aggregation, also known as tumor cell-induced platelet aggregation (TCIPA) (108, 114). PDPN-positive human glioblastoma Gli16 cells were able to markedly induce platelet aggregation, whereas not detected by PDPN-negative cells (115). In tumor-bearing mouse models, either ablating *PDPN* gene or blocking PDPN by monoclonal antibody (mAb) injection effectively suppressed platelet aggregation, supporting that PDPN is crucial for TCIPA formation (116, 117). The PDPN-mediated TCIPA was strongly associated with an increased incidence of VTE in cancer patients (115, 118). Moreover, PDPN overexpression is also involved in TCIPA-induced tumor promotion and progression. The platelet-tumor aggregates are readily arrested in the microvasculature, facilitating tumor metastasis (20). PDPN neutralization significantly inhibited TCIPA occurrence, tumor growth, and metastasis in nude mice injected with human melanoma or lung cancer cell lines (108, 116, 119). Moreover, platelet-derived TGFβ upregulated PDPN expression in human bladder cancer cells, which induced EMT process and cancer cell invasion (120). Taken together, PDPN is considered as a ‘pinpoint’ that interconnects between tumor and platelets, regulating VTE as well as tumor progression.

### Platelet CLEC2-PDPN Axis: A Pinpoint of Platelet-Tumor Cell Interaction

**Figure 1:**
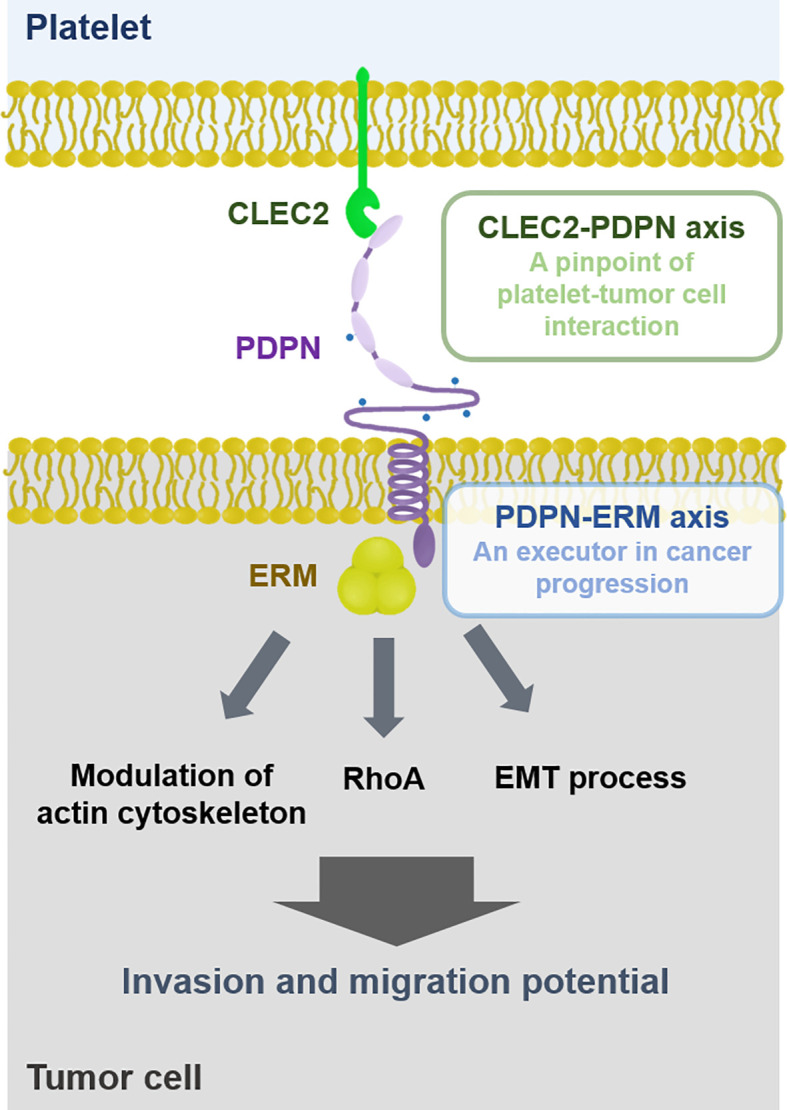
Interaction between platelet and tumor cell. Platelets can physically interact with tumor cells *via* the CLEC2-PDPN axis. PDPN is associated with ERM proteins that promote cancer cell migration and invasion through modulating actin cytoskeleton, RhoA, and EMT process. Thus, the CLEC2-PDPN-ERM axis is a crucial target for chemotherapy.

PDPN consists of an extracellular domain, a transmembrane domain, and a cytoplasmic domain (121). The extracellular domain of PDPN carries four platelet aggregation-stimulating (PLAG) domains with a plenty of potential O-glycosylation sites, crucial for interaction with platelets (121). The PLAG domain of PDPN has been reported to bind to CLEC2 that is abundantly expressed on the surface of platelets (122). Interestingly, CLEC2-deficient mice phenocopy PDPN-knockout mice, like prenatal lethality and impaired lymphatic vasculature (123). Either platelet-specific deletion of CLEC2 or inhibition of PDPN was associated with reduced thrombosis in a murine deep vein thrombosis model of inferior vena cava stenosis (124). Similarly, cancer cell lines with high endogenous PDPN expression levels, such as LN319 and Colon-26, showed induced platelet aggregation, which was attenuated by pre-incubation with an anti-CLEC2 antibody (125). Tsukiji et al. have found that cobalt hematoporphyrin (Co-HP) directly binds to PDPN-binding sites of CLEC2, functioning as an inhibitor of the CLEC2-PDPN axis (126). Both Co-HP administration and CLEC2 neutralization significantly inhibited CLEC2-dependent platelet aggregation in tumor-bearing mice (126, 127). Taken together, these data support that PDPN is interdependent with CLEC2, thus, the platelet CLEC2-PDPN axis is crucial for platelet-tumor cell interaction (**Figure 1**).

In conjunction with TCIPA formation, the platelet CLEC2-PDPN axis mediates cancer promotion and progression. In mice inoculated with PDPN-expressing B16F10 melanoma cells, CLEC2 depletion by anti-CLEC2 mAb 2A2B10 injection reduced plasma levels of inflammatory cytokines and lung metastasis, resulting in prolonged survival compared to control mice (127). Treatment with a CLEC2 inhibitor Co-HP suppressed lung metastasis of PDPN-expressing melanoma cells, but not that of PDPN-negative lung cancer cells (126). In platelet-depleted mice, platelet transfusion induced much more lung colonization as well as bone metastasis of PDPN-expressing osteosarcoma cells, while CLEC2 mAb injection reduced lung colonization (68). Likewise, injection of PDPN mAb (MS-1) remarkably suppressed platelet aggregation as well as lung metastasis in the murine cancer metastasis model (128). Therefore, the platelet CLEC2-PDPN axis is considered as a pinpoint for platelet-tumor interaction that promotes tumor progression (**Figure 1**). It has been demonstrated that CLEC2 deficiency is not significantly related to bleeding tendency (123, 129). In this regard, the platelet CLEC2-PDPN axis could be a promising target to inhibit TCIPA-induced tumor progression without bleeding risk, a major complication of the traditional antiplatelet agents.

### PDPN-ERM Axis: An Executor in Cancer Progression

PDPN has a short cytoplasmic tail associated with ezrin/radixin/moesin (ERM) proteins that primarily bridge between plasma membrane proteins and F-actin filaments of the cytoskeleton (100, 130). It is well documented that cells and tissues utilize this ERM crosslink system to maintain the architectures necessary for their own biological functions (131). In particular, ERM proteins are crucial regulators for epithelial morphogenesis and integrity, mitosis, cell polarity, and cell adhesion (132, 133). Among the ERM protein members, ezrin-null mice displayed much more severe phenotypes compared to moesin- or radixin-deficient mice (134). Ezrin-deficient mice showed defects in intestinal villus morphogenesis and epithelial cell organization (135). In addition, ERM proteins regulate the cell-cell and cell-matrix interactions, particularly in cancer cells (136). Thus, PDPN is engaged in cell adhesion, migration, and invasion through association with the ERM proteins, as illustrated in **Figure 1** (113, 133).

PDPN expression is upregulated peculiarly in the growing edge of tumors and commonly co-localized with ERM proteins (100, 106). Similar to PDPN, overexpression of ERM proteins has been detected in various types of cancers: ezrin overexpression in breast, hepatocellular, colon, ovarian, and pancreatic cancers (137–141); radixin overexpression in pancreatic cancer with lymph node metastasis (142); moesin overexpression in skin cancer, colorectal carcinoma, endometrial adenocarcinoma, and glioma (143–146). Moreover, upregulation of ERM proteins is associated with poor prognosis in cancer patients (140, 147–150). In athymic nude mice, intracranial injection of moesin-overexpressing glioblastoma cells significantly reduced the survival rates compared to the control group (146). Moreover, ERM proteins were frequently mislocalized during tumor progression, from plasma membrane to cytoplasm (136). Thus, dysregulation of ERM proteins takes part in cancer promotion and progression, possibly in an interdependent manner with PDPN.

It has been reported that PDPN mediated TCIPA-induced EMT process in human cancer cell lines (120). In non-cancerous experimental settings, PDPN can bind to ERM proteins through its cytoplasmic domain, promoting the EMT process as well as cell migration (130, 151). Silencing of radixin, one of the ERM protein members, suppressed the EMT process as well as migration and invasion in human gastric carcinoma SGC-7901 cells (152). Moreover, PDPN can induce migration ability in cancer cells that bypass the EMT process *via* filopodia formation (106). Instead of the EMT process, PDPN recruits ERM proteins to modulate the actin cytoskeleton in a RhoA-dependent manner, consequently promoting cancer cell migration and invasion. Taken together, the PDPN-ERM axis can promote migratory capability and invasiveness of tumor cells, through either EMT process or cytoskeletal rearrangement.

It has been reported that the CLEC2-PDPN axis can regulate cell contractility and migration through activation of ERM proteins in non-cancerous settings (153–155). In this regard, it is plausible that the PDPN-ERM axis could be recruited by tumors bound to platelets *via* CLEC2-PDPN interaction, conferring cancer cells metastatic potentials (**Figure 1**). Further investigation is necessary to clarify the role of the platelet CLEC2-PDPN-ERM axis in cancer progression.

### Platelet CLEC2-PDPN and PDPN-ERM Axes in OSCC

According to the Cancer Genome Atlas analysis, head and neck cancer patients present much higher PDPN expression levels compared to other types of cancer patients. While PDPN expression is rarely detected in normal oral epithelial cells, OSCC patients show upregulation of PDPN in tumors, which contributed to poor prognosis (156–159). In the xenograft mouse model, PDPN-overexpressing OSCC cells promoted tumor growth and intratumoral platelet accumulation, implying that PDPN mediates TCIPA formation in OSCC (160). Similar to high platelet counts (90), elevated PDPN expression was often found at the invasive front and correlated with lymph node metastasis in OSCC patients (156, 161). In line with that, silencing of *PDPN* gene expression attenuated migration and invasion in human OSCC cell lines (160, 162–164). Considering that platelet CLEC2 is crucial for PDPN-dependent TCIPA formation, the platelet CLEC2-PDPN axis would be a feasible target for successful local control in OSCC patients.

In OSCC patients, overexpression of ezrin and moesin has been detected in advanced staged tumors and significantly associated with worse overall survival rates (149, 164, 165). Kobayashi et al. have reported that cytoplasmic expression of moesin shows a strong correlation with lymph node metastasis in OSCC patients (166). Of note, PDPN expression was positively related to ezrin expression, particularly in the cytoplasm of the odontogenic tumors (167). Moreover, this co-expression between PDPN and ezrin was frequently detected in the invasive front and possibly involved with lymph node metastasis in the lip cancer (168). These data suggest that the PDPN-ERM axis may contribute to increased metastatic potential in OSCC. In consistent, PDPN has been reported to enhance cell motility and invasiveness through interaction with ERM binding partners, such as membrane type 1 MMP, Cdc42, and CD44, in humans OSCC cell lines (162, 164). These data suggest that ERM proteins function as an intracellular executor of the CLEC2-PDPN axis in invasion and metastasis of OSCC.

## Targeting Platelet-Tumor Interaction for Chemotherapy

### Aspirin

Considering pro-tumorigenic activities of platelets, antiplatelet agents could be promising chemotherapeutics, as shown in **Table 1**. A classical antithrombotic drug aspirin has been used for chemoprevention. The meta-analysis and retrospective cohort study showed that a regular use of aspirin is associated with reduced risk of cancers in liver, stomach, colorectum, lung, pancreas, and oesophagus (26, 169). In head and neck cancer patients, evaluation of aspirin as a chemopreventive agent is still controversial. A hospital-based case control study revealed that aspirin use can reduce head and neck cancer risk (170), whereas the other investigations demonstrated that there was no significant correlation between aspirin intake and head and neck cancer (171, 172). Moreover, the risk of gastrointestinal bleeding could limit the use of aspirin for cancer prevention and/or treatment (29, 30).

**Table 1:** Strategies to target platelet-tumor interaction for chemotherapy.

Agent	TCIPA	Cancer risk/metastasis	Bleeding	References
**Classical antiplatelet drug**				
Aspirin	Inhibit TCIPA *in vitro* and *in vivo*	Inhibit metastasis *in vivo*	Increased gastrointestinal bleeding	(26, 29, 30, 169–175)
		Cancer preventive effect in human subjects (controversial in head and neck cancer)		
		Reduce metastasis in cancer patients		
**P2Y12 receptor antagonism**				
Clopidogrel	Inhibit TCIPA in mice	Inhibit tumor metastasis in mice	A long-term use can increase bleeding risk	(176–182)
		No impact on cancer motility in human colorectal, breast, and prostate cancer patients		
Ticagrelor	Inhibit TCIPA	Increase cancer risks in human	More major bleeding compared to clopidogrel in patients with acute coronary syndrome	(180, 183–185)
**GPVI antagonism**				
Anti-GPVI mAb (JAQ1)	Inhibit TCIPA	Inhibit cancer cell extravasation *in vitro*	No impact on bleeding time	(129, 186–188)
		Inhibit metastasis in mice		
		Induce intratumoral hemorrhage and accumulation of co-administrated anticancer drugs in mice		
Revacept	Inhibit TCIPA in mice and human	Inhibit EMT marker expression *in vitro*	No impact on bleeding time in mice and human	(189–191)
**Targeting CLEC2-PDPN axis**				
Anti-CLEC2 mAb (2A2B10 and INU1)	Inhibit intratumoral thrombus formation in mice	Inhibit metastasis in mice	No impact on bleeding time	(68, 127, 129)
Anti-PDPN mAb (NZ-1, MS-1, and SZ-168)	Inhibit platelet aggregation in mice	Inhibit metastasis in mice		(119, 128, 192–194)
		Inhibit VET in mice		
2CP	Inhibit TCIPA in mice	Inhibit metastasis in mice	No impact on bleeding time	(195)
Co-HP	Inhibit platelet aggregation	Inhibit metastasis in mice	No impact on bleeding time	(126)
		Inhibit VET in mice		
Polysaccharide extracted from *Artemisia argyi* leaves	Inhibit TCIPA			(196)

### Platelet P2Y12 Receptor Antagonists

**Figure 2:**
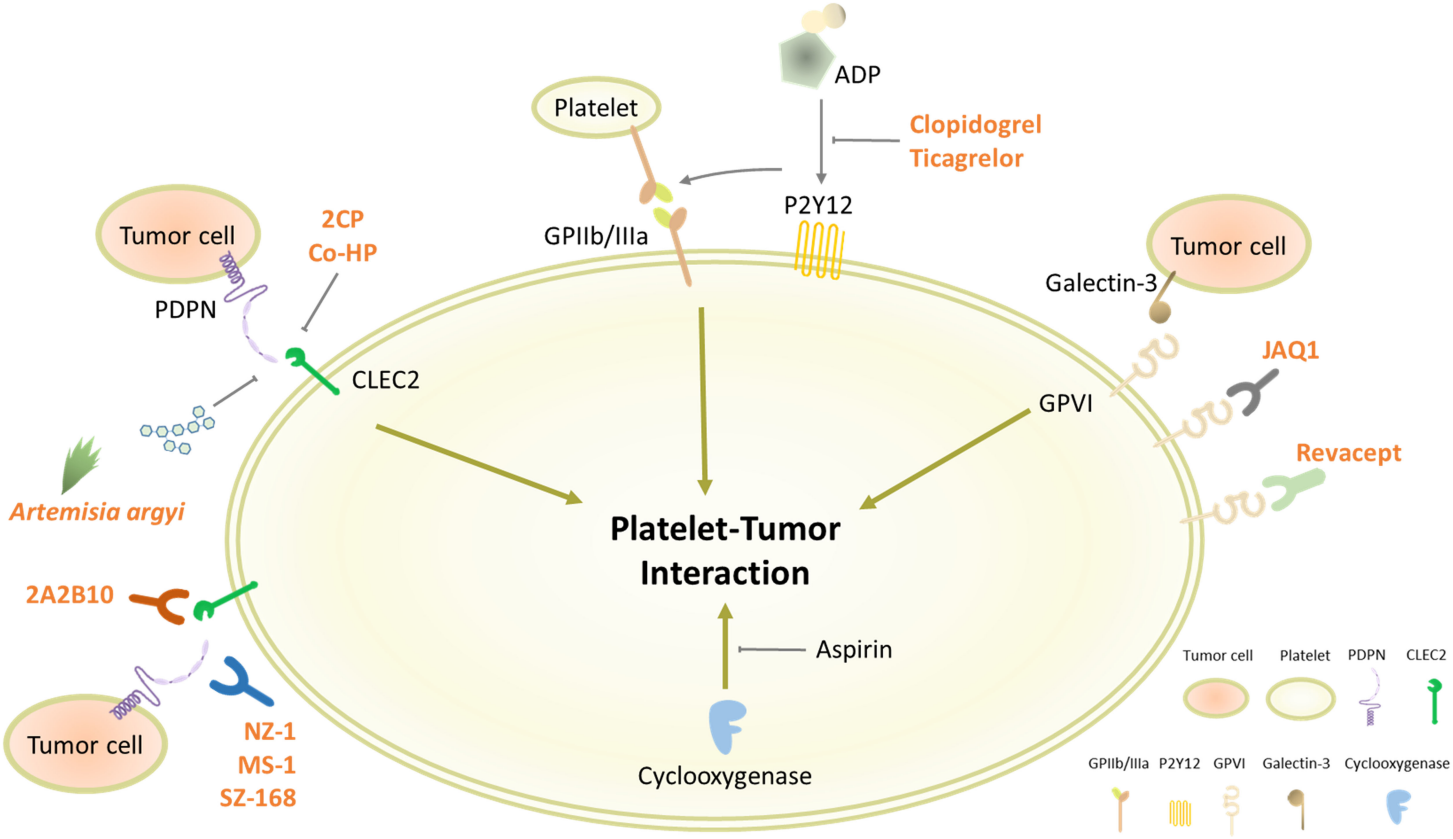
Platelet receptors involved in platelet-tumor interaction. Platelets contain various types of receptors on the cell surface for diverse physiological functions, including cell adhesion and aggregation. Some of the surface molecules, such as CLEC2, P2Y12, and GPVI, can promote the interaction between platelets and cancer cells, which could be plausible targets for blocking TCIPA formation.

Platelet P2Y12 receptor is involved in ADP-stimulated activation of glycoprotein IIb/IIIa (GPIIb/IIIa) responsible for platelet aggregation (197). It has been reported that GPIIb/IIIa mediates platelet-tumor interaction and cancer metastasis (198–200). In conjunction with GPIIb/IIIa, stimulation of P2Y12 receptor can promote platelet-tumor crosstalk and cancer metastasis (**Figure 2**), suggesting P2Y12 receptor antagonists as anticancer drugs (73, 201). Clopidogrel, the most widely used P2Y12 receptor antagonist, markedly inhibited tumor growth in mouse ovarian and liver cancer models (176, 177). Another P2Y12 inhibitor ticagrelor suppressed proliferation of ovarian cancer cells *in vivo* and *in vitro*, which was not detected in absence of platelets (176). Moreover, treatment with ticagrelor attenuated TCIPA formation and cancer metastasis in the murine experimental models (178–180). These pre-clinical data suggest platelet P2Y12 receptor as a target for cancer treatment by controlling platelet-tumor aggregation. However, a population-based cohort study showed that the use of clopidogrel has no huge impact on cancer mortality in colorectal, breast, and prostate cancer patients (181). Even worse, the clinical trial-based analyses revealed that ticagrelor increased cancer risks (183, 184). In another patient-level meta-analysis of randomized clinical trials, a long-term intake of clopidogrel was associated with bleeding risk and hemorrhage (182). Overall, the use of P2Y12 receptor antagonists for chemotherapy is controversial, in spite of the compelling pre-clinical evidence.

### Platelet GPVI Antagonism

GPVI is the major platelet-activating receptor exclusively expressed on platelets and megakaryocytes (202). GPVI-null mice showed lack of thrombus formation and defective platelet activation without severe bleeding tendency (203, 204). Moreover, these GPVI-deficient mice developed less metastatic tumors by injection of lung cancer or melanoma cells than WT mice (205). Notably, platelet GPVI can bind to galectin-3 on tumor cells, provoking platelet-tumor cell interaction and metastasis (**Figure 2**) (186, 189). These pre-clinical data suggest that GPVI antagonism is a conceivable strategy to block TCIPA-mediated tumor progression without adverse effects. In line with this notion, platelets preincubated with an anti-GPVI antibody (JAQ1) were less able to form aggregates with human breast cancer cells and eventually reduced cancer cell extravasation in the transendothelial migration assay (187). Moreover, treatment with JAQ1 reduced tumor metastasis in the murine lung metastasis models, further supporting antitumor effects of GPVI antagonism *via* blocking TCIPA formation (186). Interestingly, JAQ1 Fab2 fragment induced intratumoral hemorrhage that led to accumulation of co-administrated chemotherapeutics without systemic bleeding complications, thus allowing to maximize anticancer effects (188). Revacept, a competitive GPVI inhibitor comprising a soluble Fc fusion protein, decreased platelet-tumor interaction and metastatic potential *in vitro* (189). In atherosclerotic mice and healthy human subjects, Revacept reduced platelet aggregation with no impact on bleeding times (190, 191). Based on this drug safety assurance, the antitumor efficacy of GPVI antagonists must be further evaluated in human cancer patients.

### Targeting Platelet CLEC2-PDPN Axis

As described in **Figure 1**, the platelet CLEC2-PDPN axis is emerging as a pinpoint to control the platelet-tumor interaction and subsequent tumor progression. In order to disconnect the platelet CLEC2-PDPN axis, diverse approaches have been made, including mAbs against CLEC2 or PDPN and pharmacological inhibitors. PDPN mAbs, such as NZ-1 and MS-1, can bind to the PLAG domain of PDPN and neutralize interaction with platelet CLEC2 (**Figure 2**) (192, 193). These PDPN mAbs specifically inhibited PDPN-mediated platelet aggregation and cancer metastasis in the murine experimental models (128, 192, 193). Moreover, anti-PDPN antibody SZ-168 reduced the incidence of VTE in mice (194). Similar to PDPN mAbs, anti-CLEC2 antibody 2A2B10 suppressed intratumoral thrombus formation as well as metastasis in mice (68, 127). These investigations suggest that mAbs neutralizing either CLEC2 or PDPN specifically inhibit platelet-tumor interaction and tumor metastasis. Although the influence of CLEC2 deficiency on bleeding is conflicting in CLEC2-null mice, CLEC2 mAb-treated mice had no sign of prolonged bleeding compared to control mice (123, 129, 206, 207). Overall, CLEC2 neutralization seems not to affect bleeding time profoundly.

In addition to neutralizing antibodies, pharmacological inhibitors display potent inhibitory effects on the CLEC2-PDPN axis. Chang et al. have newly synthesized a non-cytotoxic 5-nitrobenzoate compound 2CP that specifically inhibits the CLEC2-PDPN interaction (195). 2CP selectively blocked PDPN-induced TCIPA formation and lung metastasis in the xenograft model, whereas bleeding time was not affected by 2CP (195). Co-HP can directly bind to CLEC2 at PDPN-binding sites and potently block CLEC2-PDPN interaction (126). Co-HP injection significantly reduced tumor metastasis and the incidence of VTE in mice, but not affecting the bleeding time (126). Moreover, a bioactive polysaccharide extracted from *Artemisia argyi* leaves inhibited CLEC2-PDPN interaction and PDPN-dependent TCIPA formation (196).

Taken together, inhibition of the platelet CLEC2-PDPN axis is a promising chemotherapeutic strategy by suppressing TCIPA formation and metastasis (**Table 1**). In particular, targeting the CLEC2-PDPN axis seems to be a relatively safer approach to block platelet-tumor interaction without severe adverse effects, such as increased bleeding risk. Further clinical studies are needed to validate their anti-thrombotic and anti-metastatic effects in human subjects. Although targeting the CLEC2-PDPN axis is relatively harmless, it still requires caution to be clinically applied, since CLEC2- or PDPN-deficient mice showed abnormal lymphatic vessel formation (123).

## Conclusion

Despite advances in surgical techniques and therapeutic strategies including radiotherapy and immunotherapy, the survival rate of OSCC has not been improved for the past decade due to failure of local control of primary tumor (2, 208). Currently, platelets are well recognized as a stromal member of the TME and an important prognostic index in OSCC patients (25, 57, 89). In particular, platelets directly interact with cancer cells *via* CLEC2-PDPN binding, fortifying metastatic potentials of cancer cells. Regarding that PDPN is the only known endogenous ligand for CLEC2, the platelet CLEC2-PDPN axis is a pinpoint target to control TCIPA formation-mediated metastasis without undesirable complications. Thus, blockade of the CLEC2-PDPN axis could be a prospective strategy for successful local control and improvement of survival in OSCC patients, which merits further pre-clinical and clinical investigations.

## Author Contributions

N-YS contributed to study conception. S-YP, B-OH, ESC, XZ, SKL, H-JA, K-SC, W-YC, and N-YS performed literature review and analysis and revised the manuscript. N-YS, S-YP, and B-OH drafted the manuscript, figures, and tables. All authors contributed to the article and approved the submitted version.

## Funding

This research was supported by National Research Foundation of Korea (NRF) Grants funded by the Korean Government (grant numbers NRF-2020R1C1C1003338 and NRF-2016R1A5A2008630 to N-YS) and by the Yonsei University Research Fund of 2021 (Yonsei Signature Research Cluster Program 2021-22-0017).

## Conflict of Interest

The authors declare that the research was conducted in the absence of any commercial or financial relationships that could be construed as a potential conflict of interest.

## Publisher’s Note

All claims expressed in this article are solely those of the authors and do not necessarily represent those of their affiliated organizations, or those of the publisher, the editors and the reviewers. Any product that may be evaluated in this article, or claim that may be made by its manufacturer, is not guaranteed or endorsed by the publisher.
